# Molecular typing and prognostic risk models for ovarian cancer: a study based on cell differentiation trajectory

**DOI:** 10.3389/fcell.2023.1131494

**Published:** 2023-08-31

**Authors:** Tingfeng Chen, Tingting Ni, Lan Mu, Zhou Ying, Hanqun Zhang, Zi Wang

**Affiliations:** ^1^ Department of Oncology, Guizhou Provincial People’s Hospital, Guiyang, China; ^2^ State Key Laboratory of Biotherapy, West China Hospital, Sichuan University, Chengdu, China; ^3^ Department of Medical Records and Statistics, Guizhou Provincial People’s Hospital, Guiyang, China

**Keywords:** ovarian cancer, molecular typing, single cell, cell differentiation trajectory, bioinformatics

## Abstract

Ovarian cancer is a heterogeneous disease with different molecular phenotypes. We performed molecular typing of ovarian cancer using cell differentiation trajectory analysis and proposed a prognostic risk scoring model. Using the copy number variation provided by inferCNV, we identified malignant tumor cells. Then, ovarian cancer samples were divided into four subtypes based on differentiation-related genes (DRGs). There were significant differences in survival rates, clinical features, tumor microenvironment scores, and the expression levels of ICGs among the subtypes. Based on nine DRGs, a prognostic risk score model was generated (AUC at 1 year: 0.749; 3 years: 0.651). Then we obtained a nomogram of the prognostic variable combination, including risk scores and clinicopathological characteristics, and predicted the 1-, 3- and 5-year overall survival. Finally, we explored some issues of immune escape using the established risk model. Our study demonstrates the significant influence of cell differentiation on predicting prognosis in OV patients and provides new insights for OV treatment and potential immunotherapeutic strategies.

## 1 Introduction

Globally, ovarian cancer is the seventh most common cancer in women. There are approximately 225,000 new cases each year, with a survival rate of approximately 30%, ranking first in the mortality rate of gynecological malignancies. Malignant epithelial neoplasms are the most common type of ovarian cancer, accounting for nearly 90% of ovarian cancers (Chen et al., 2016; Fitzmaurice et al., 2017). Recent molecular studies have shown that epithelial ovarian cancer is a heterogeneous disease, reflected by different histological types. The heterogeneity of biological behavior is important for patient prognosis and treatment, especially for future individualized therapies. With the continuous development of advanced technologies such as genomics and proteomics, molecular targeted therapy based on molecular typing of malignant tumors has been successful in chronic myeloid leukemia, breast cancer with ER (+) or Her2 (+), and lung cancer with EGFR mutation (Roode et al., 2015; Miller et al., 2017a; Dolly et al., 2017; Xu et al., 2017), which has significantly improved the survival rate of patients.

To identify heterogeneity, previous studies have made much effort in the molecular typing of ovarian cancer. For example, in 2008, Tothill et al. (2008) determined six subtypes by K-means through miRNA gene expression profile chip detection. Tan et al. (2013) identified five molecular subtypes through functional genomics. In terms of clinical application, Kommoss et al. (2017) evaluated the relationship between TCGA molecular subtypes and the efficacy of randomly assigned beizumab, and the results showed that the median PFS of the molecular subtypes with the worst survival (proliferation and mesenchymal) was improved, but OS was not significantly changed. There is still room for molecular typing to make a more accurate diagnosis, treatment and survival prediction.

Compared to bulk RNA sequencing (RNA-seq) techniques, single-cell RNA-seq (scRNA-seq) can characterize genetic complexity at the single-cell resolution, including copy number variation (CNV), gene expression level, and gene fusion, which paves the way for us to understand the heterogeneity of cells. In this study, we performed cell typing and copy number variation analysis on the samples using scRNA-seq technology. The differentiation trajectory of OV cells was also studied. Based on this and combined with a large amount of bulk RNA-seq data, the relationship between OV cells and clinical results was studied, providing new insights for OV diagnosis and potential immunotherapeutic strategies.

## 2 Materials and methods

### 2.1 Acquisition and processing of single-cell RNA-seq

Raw scRNA-seq data from four epithelial ovarian cancer samples (GSM3729170, GSM3729171, GSM3729172 and GSM3729173) were downloaded from Gene Expression Omnibus (GEO) (dataset GSE130000) (https://www.ncbi.nlm.nih.gov/geo). The data were then processed in R version 4.0.2 (R Foundation for Statistical Computing, Vienna, Austria) using the Seurat package. The proportion of mitochondrial genes was then calculated, and its relationship with total gene numbers and sequencing depth was determined by correlation analysis. Cells in which <100 genes or with a mitochondrial gene proportion of >10% were excluded from analysis. Genes detected in less than three cells were also excluded from analysis. Each sample was normalized to concentrate the expression data with a large degree of dispersion, and the top 1,500 genes with significant differences across cells were identified by variance analysis. In the normalization process, we first calculate the size factor by dividing the total gene UMI count in the cell by a scale factor of 10,000. Then the UMI count is divided by the cell size factor to obtain the normalized UMI count for each gene. Finally, take the normalized UMI count as the log of 2. Then we selected the common characteristic genes of different samples and integrated them. We used a harmony integration algorithm to preserve biological variation and the continuous state of developmental cells while reducing experimental and technical batch effects, rather than erroneously clustering cells into discrete groups (Korsunsky et al., 2019).

### 2.2 Dimensionality reduction and cell annotation

We used the “ScaleData” function in the Seurat R package to scale the matrix. The Article “FindVariableGenes” and “RunPCA” functions were used to identify highly variable genes, which could preserve major biology variation. Then we used principal component analysis (PCA) for dimensionality reduction. Based on the PCA results, an appropriate number of principal components were used. Using the “FindNeighbors” and “FindClusters” functions, we determine an optimal number of cell clusters for further unsupervised graph-based clustering. Uniform Manifold Approximation and Projection (UMAP) algorithm with a resolution of 0.4 was used to show the main cell clusters. Under the condition of log_2_(Fold Change) > 0.25 and False Discovery Rate (FDR) < 0.01, marker genes were screened out through the function “FindAllMarkers” in Seurat with default parameters of the Wilcoxon rank-sum test. Visualize the marker genes in each cluster using the ggplot2 package in R. Using the marker genes of each cell type summarized in Kan’s study (Kan et al., 2022), together with the EnrichR database (Chen et al., 2013; Kuleshov et al., 2016; Xie et al., 2021), we determine the marker genes. Based on expression of obtained marker genes and the top 50 most upregulated genes in each cluster, cell clusters were finally annotated.

### 2.3 InferCNV analysis

DNA copy number variation (CNV) has been recognized as an important source of genetic variation. By using the inferCNV (https://github.com/broadinstitute/infercnv) package in R (Navin et al., 2011), we calculated somatic large-scale chromosomal CNVs, such as gains or deletions of entire chromosomes or large segments of chromosomes, in each single cell to identify malignant epithelial cells. InferCNV sorted all analyzed genes by their genomic locations and applied a moving average of 101 genes. We downloaded the human genome assembly GRCh38 from NCBI and prepared a gene/chromosome position file.

Endothelial cells and T cells were selected as reference normal cells. The inferCNV used a Hidden Markov Model (HMM) Model (i6 HMM model) to predict CNV level and implemented a Bayesian Network Latent Mixture Model to identify the posterior probabilities of alteration status in each cell and whole CNV region to correct the results. The i6 HMM model was a six-state CNV score model to predict the following CNV levels: 0: complete loss; 0.5: loss of one copy; 1: neutral; 1.5: addition of one copy; 2.0: addition of two copies; 3.0: >2 copies. Based on the CNV score, we generated a heatmap. The result showed that epithelial cells were labeled as malignant tumor cells, while CAFs and macrophages were labeled as non-tumor cells.

### 2.4 Single-cell pseudotime and trajectory analysis

It is known that single-cell trajectories can unveil how gene regulation governs cell fate decisions. We found Most cell-state transitions, whether in development, reprogramming, or disease, are characterized by cascades of gene expression changes. We used a technique called “pseudotemporal ordering,” which applies machine learning to single-cell transcriptome sequencing (scRNA-seq) data to order cells along a reconstructed “trajectory” of differentiation or other type of inferred biological transition. By using the Monocle package in R (Navin et al., 2011), we performed pseudotime and trajectory analyses of ovarian cancer cells.

We convert Seurat results to the cell matrix, cell annotation table and gene annotation table required by monocle. To create a CellDataSet object with parameter “expressionFamily = negbinomial.size(),” we used “newCellDataSet” function in the Monocle package.

Dimensionality reduction was performed using the DDRTree algorithm. The cell lineage trajectory based on cell cluster and pseudotime was then inferred with the default parameters of Monocle after dimensionality reduction and cell ordering, then visualized with the “plot_cell_trajectory” function. Following cell trajectory, The cells were split into different subsets. Using the “FindMarkers” function, Intracellular differentially expressed genes in cells were identified as differentiation-related genes (DRGs) with |log_2_(FC)| >0.25 and FDR < 0.05.

### 2.5 Acquisition and processing of bulk RNA-seq

Raw bulk RNA-seq data and survival data from 380 epithelial ovarian cancer samples were downloaded from Gene Expression Omnibus (GEO) (dataset GSE140082). Most of them are serous, accounting for 73% of the total. The rest are referred to as “other” and we excluded them because the histological type was unclear. From The Cancer Genome Atlas (TCGA), we downloaded 379 OV samples with transcriptomic data and clinical data. They’re all serous epithelial ovarian cancer. These with survival <30 days or >2,000 days and with unclear survival status or clinicopathological characteristics were excluded in this study.

### 2.6 DRGs-based molecular subtypes of OV patients

To identify molecular subtypes, unsupervised consensus clustering was performed to cluster ovarian cancer samples into subtypes based on the expression matrix of DRGs using R’s ConsensusClusterPlus package (Wilkerson and Hayes, 2010). The following parameters were used for clustering: number of repetitions = 50 bootstraps; pItem = 0.8 (resampling 80% of any sample); pFeature = 1 (100% of features to sample) and clustering algorithm = k-means method. Set random seed “123,456” for reproducible results. The cumulative distribution function (CDF) method was used to determine the optimal number of subtypes.

The clustering results were then intersected with the clinical data. Data with missing survival information were excluded from the analysis. Using “survdiff” in the survival package, we performed Kaplan–Meier analysis to obtain survival differential statistics. This was then visualised using the ggsurvplot function.

### 2.7 Tumor microenvironment scores, immune checkpoint genes expression across clusters

Cells in the tumor microenvironment and the extent of infiltrating immune and stromal cells in the tumor are important contributors to prognosis. Immune and stromal cells are two major types of non-tumor components in the tumor microenvironment, which have been proposed to be valuable in the diagnosis and prognostic assessment of tumors (Gajewski et al., 2013).

The immune and stromal scores calculated based on the ESTIMATE algorithm can facilitate the quantification of immune and stromal components in tumors. In this algorithm, immune and stromal scores are calculated by analysing specific gene expression characteristics of immune and stromal cells to predict the infiltration of non-tumor cells. We used function “estimateScore” in ESTIMATE package, for sample interstitial, immune, and tumor purity scores. When stromal cells and immune cells are high, tumor purity will be low and conversely, tumor purity will be high.

In addition, 38 ICGs (immune checkpoint genes) were collected from previous studies (Patel et al., 2017; Garris et al., 2018; Zhang et al., 2018; Wang et al., 2019a; Wang et al., 2019b; Han et al., 2019; Xiang et al., 2021). Immune checkpoints are a set of molecules expressed on immune cells that can regulate the level of immune activation and play an important role in preventing autoimmune dysfunction. The expression of these genes in different clusters was evaluated. Kaplan–Meier survival analysis was used to determine the prognostic value.

### 2.8 Generation and quality evaluation of prognostic risk scoring models

In our study, the TCGA cohort and GSE140082 dataset were used as the training and validation sets of the risk scoring model. We extracted the expression levels of DRGs in GEO and TCGA cohorts and normalized the expression matrix using log2 transformation. Weighted correlation network analysis (WGCNA) was performed in TCGA queues, and correlations between key modules and OV differentiation were determined. The function “pickSoftThreshold” is used to find the soft threshold and to construct the scale-free distribution network. Then the functions “cutreeDynamic,” “moduleEigengenes,” “mergeCloseModules” are used to cluster and merge the modules. A heat map of the correlation between modules and clinical features was drawn.

Univariate analysis was performed for the genes in the key modules based on prognostic correlation (*p* < 0.05). We mapped the forest plot of single factor significant genes. The selected genes were then subjected to multivariate Cox regression analysis to generate a model for DRGs based on prognostic risk score (RS) characteristics. We used risk scores to divide the samples into high-risk and low-risk groups. Then we used Kaplan–Meier for survival analysis and receiver operating characteristic (ROC) curves to assess model accuracy and predictive efficiency.

### 2.9 Nomogram construction of TCGA cohort

A nomogram was constructed for the TCGA cohort, including RS and clinical variables such as age, grade and stage. After univariate and multivariate analysis, we obtained a nomogram of the prognostic variable combination. The relationship between these variables and survival is shown visually. Then using the “cph” function in the rms package, we predicted the 3-year and 5-year overall survival. The nomogram was tested using calibration curves which assess the predictive validity and accuracy of the nomogram.

### 2.10 GO enrichment analysis and GSVA

By using the “enrichplot,” “ggplot2,” “ClusterGVis,” and “clusterProfiler” packages in R, which can help the process of biological-term classification and visualization, GO pathway enrichment analysis was performed. We converted the gene ID through the database “org.hs.eg.db.” To investigate differences in biological states and pathways between different cell types, we used Gene Set Variation Analysis (GSVA), a non-parametric and unsupervised analysis method used to evaluate the gene set enrichment results of biological states. The main purpose is to evaluate whether different metabolic pathways are enriched in different samples by converting the expression matrix of genes between different samples into the expression matrix of gene sets between samples. The “GSVA,” “GSEABase,” and “limma” packages were used for GSVA analysis (Hänzelmann et al., 2013). The gene set database was downloaded from the Molecular Signatures Database (MSigDB) (Mootha et al., 2003; Subramanian et al., 2005). Single-cell samples were divided into high and low risk groups according to the risk scoring model. The pathway of enrichment difference between high and low risk group was found by difference analysis.

## 3 Results

### 3.1 Quality control, integration and normalization of scRNA-seq

**FIGURE 1:**
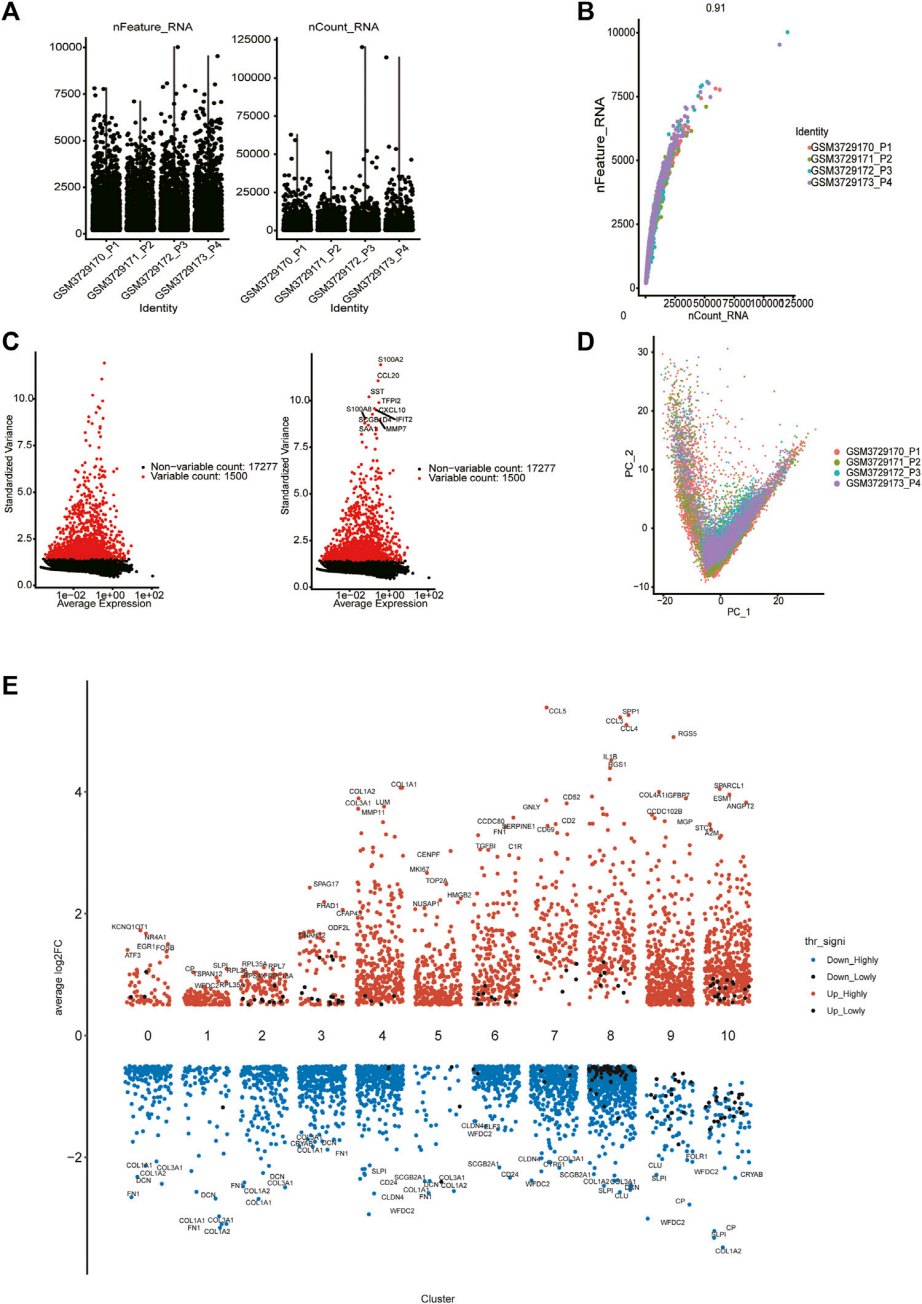
Single cell data quality control and principal component analysis. **(A)** Upon quality control and lognormalize normalization, 6,037 cells from four ovarian cancer samples remained. **(B)** A significant positive correlation between sequencing depth and intracellular total sequence. **(C)** A total of 18,777 genes were included, 1,500 variable genes had high variation. **(D)** PCA based on integrated samples, cell coordinates fit as well as possible. **(E)** Expression analysis of marker genes in each cluster (from 0 to 10).

From the GSE130000 dataset, four primary ovarian cancer samples were selected for subsequent analysis, with a total of 13,447 cells involved. Cells with fewer than 100 genes or more than 10% mitochondria were filtered out (Figure 1A). There was a significant positive correlation between sequencing depth and intracellular total sequence was detected (Figure 1B). For each sample, we selected 1,500 high variation genes. Based on the characteristic anchor genes, we integrated samples from four different patients to remove batch effects.

### 3.2 Five cell types were annotated

**FIGURE 2:**
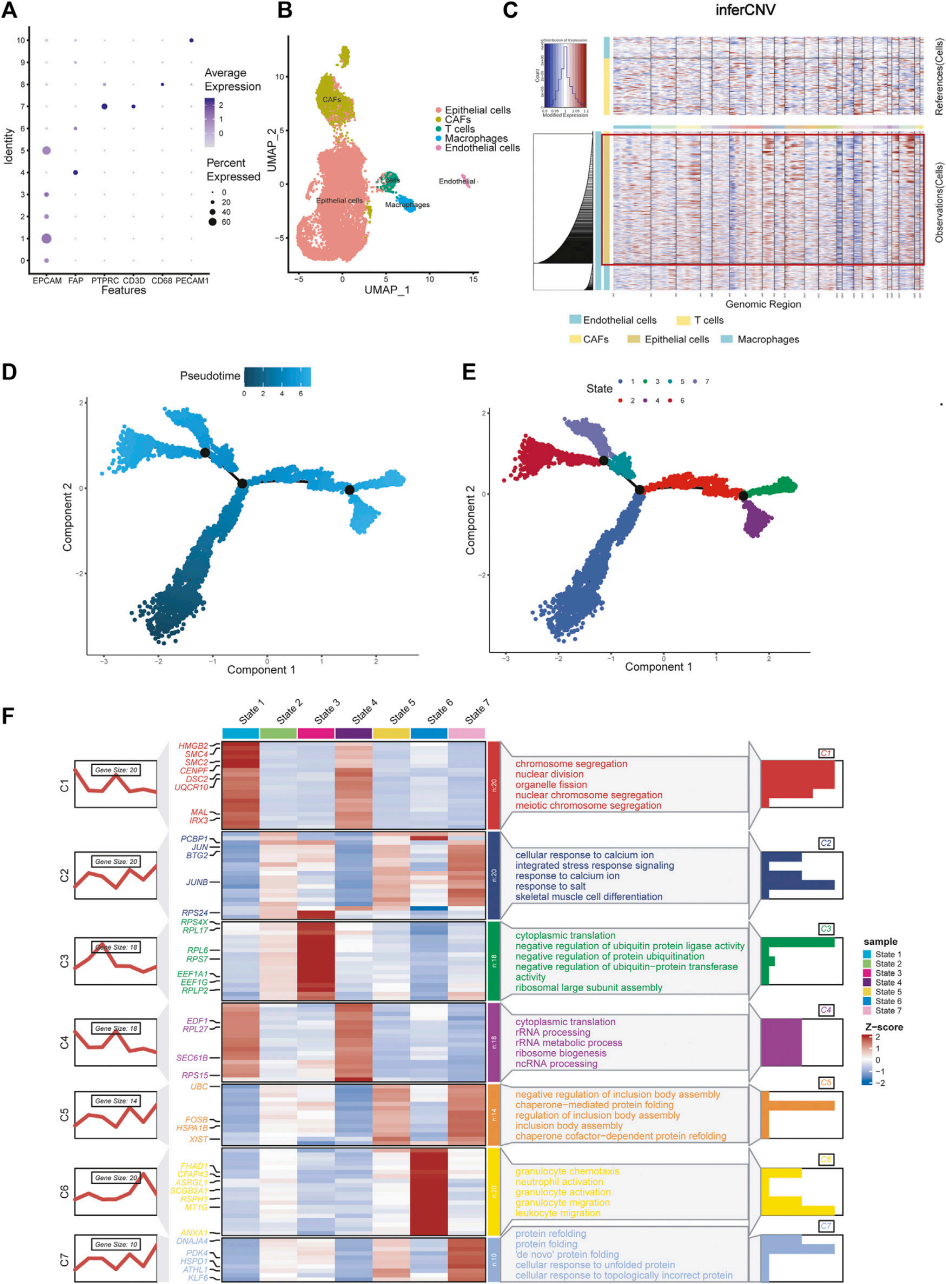
Celltype annotions, inferCNV and cell trajectory difference analysis. **(A)** Marker genes used to identify cell types in different clusters. **(B)** Eleven clusters were annotated and performed on UMAP. **(C)** InferCNV analysis showing copy number variation in epithelial cells, copy number variation in epithelial cells was significantly higher than that in other cells. **(D)** Pseudotime and cell trajectory analysis. **(E)** We identified seven states of differentiation in ovarian cancer epithelial cells. **(F)** Marker gene heat map and pathway enrichment analysis of seven different cell states.

We selected the top 30 principal components and the top 1,500 variable genes (Figure 1C) to use principal component analysis (PCA). The cell coordinates of different samples on the projection map were close, which indicated that the integration effect was great (Figure 1D). Using the Uniform Manifold Approximation and Projection (UMAP) algorithm with a resolution of 0.4, 11 main cell clusters were classified. Based on preferentially or uniquely expressed marker genes in each cluster (Figure 1E), the EnrichR datasets and the marker genes of cell type provided by T. Kan et al. (Korsunsky et al., 2019), we annotated the clusters. We also show marker genes used to identify cell types in different clusters (Figure 2A). Clusters 0, 1, 2, 3, and 5 were all epithelial cells, cluster 7 was T cell, clusters 4, 6 and 9 were CAFs (cancer-associated fibroblasts), cluster 8 was macrophage and cluster 10 was endothelial cell (Figure 2B).

### 3.3 InferCNV analysis showing copy number variation in epithelial cells

There are two methods for identifying epithelial tumor cells: one is based on copy number changes and point mutations, and the other is based on the expression of epithelial markers (Tirosh et al., 2016; Puram et al., 2017). Puram et al. (2017) demonstrated consistency between the two approaches. Kan et al. (2022) identified epithelial cells as malignant tumor cells by analysing epithelial markers. Here, we reproduced the results using InferCNV analysis. The results showed that copy number variation in epithelial cells was significantly higher than that in other cells, which verified the conclusion (Figure 2C).

### 3.4 Cell trajectory analysis identified DRGs

Using pseudotime and trajectory analysis, we identified seven different states of differentiation in ovarian cancer cells, which are epithelial cells. We defined their differential genes as differentiation-related genes (DRGs) for subsequent experiments (Figures 2D, E). We showed the marker genes for each state with a heat map and a line map. We also performed GO pathway enrichment analysis for each state (Figure 2F). State 1 mainly controls chromosome separation and nuclear division, which means the initial state of differentiation of the cells. State 2 is enriched to integrated stress response signaling and other response signaling pathways, which usually used by cells to respond to various adverse stimuli. State 3 is mainly enriched to negative regulation of protein. State 4 is related to cytoplasmic translation and rRNA processing, which may be an intermediate process. State 5 corresponds to some of the pathways involved in the formation of protein structures. State 6 is characterized by a slight increase in granulocyte behavior relative to other epithelial cancer cells. State 7 is associated with protein folding, and cells may further proliferate.

### 3.5 Four DRGs-based molecular subtypes

**FIGURE 3:**
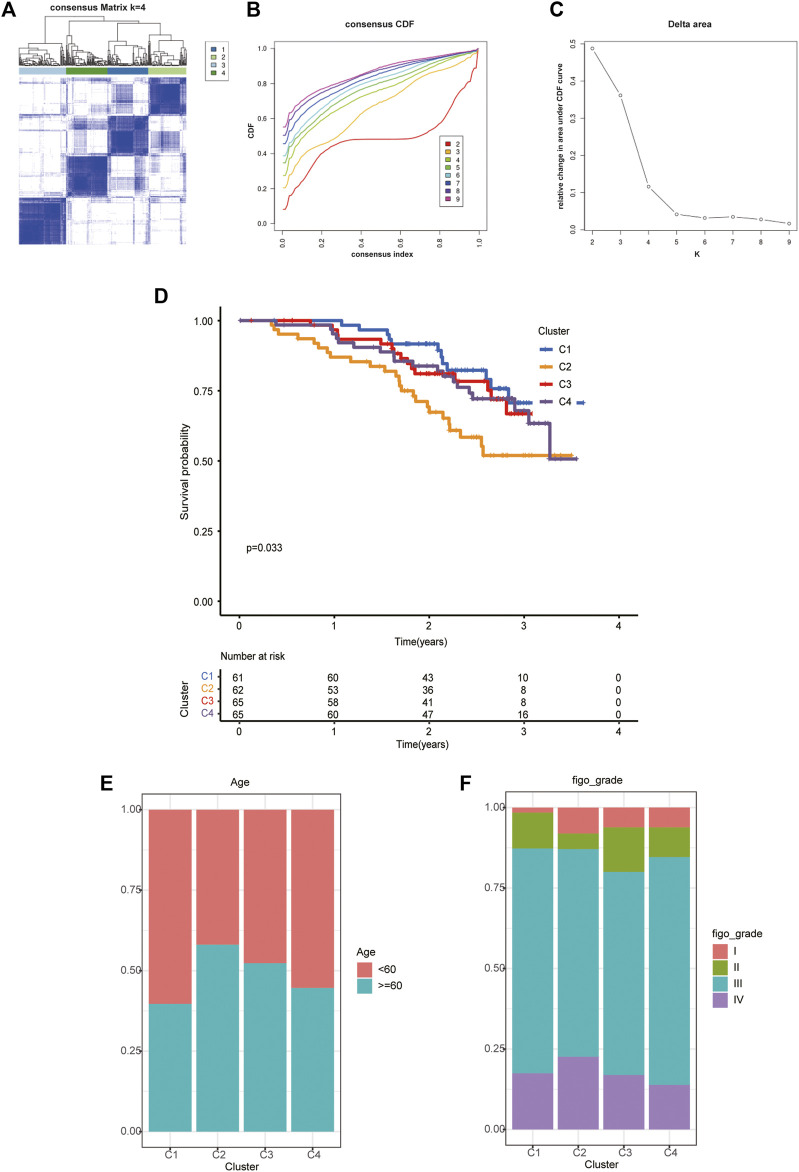
The four distinct subtypes identified and feature analysis. **(A)** Unsupervised consensus clustering was performed to cluster ovarian cancer samples into subtypes. **(B)** The cumulative distribution function (CDF) method was used to determine the optimal number of subtypes. **(C)** Relative change in area under CDF curve. Finally, four OC molecular subtypes were identified. **(D)** Kaplan-Meier analysis of the survival rates associated with the four subtypes. **(E)** The age of patients associated with the four subtypes. **(F)** The age of patients associated with the four subtypes.

With a clustering threshold of maxK = 9, we completed DRG-based consensus clustering on the GSE140082 dataset. The OV samples were grouped into four molecular subtypes (Figures 3A–C). Then, we performed Kaplan–Meier analysis on the survival rates associated with the four subtypes. The results showed that subtype I (C1) had the best OS (overall survival), followed by subtype III (C3) and subtype IV (C4), and subtype II (C2) had the worst OS (Figure 3D).

Based on the analysis of clinical data, we found that in different subtypes, with the decrease in survival probability, the age of patients tended to increase (Figure 3E), and the FIGO classification also tended to be later (Figure 3F).

### 3.6 Analysis of tumor microenvironment scores and ICGs expression across OV clusters

**FIGURE 4:**
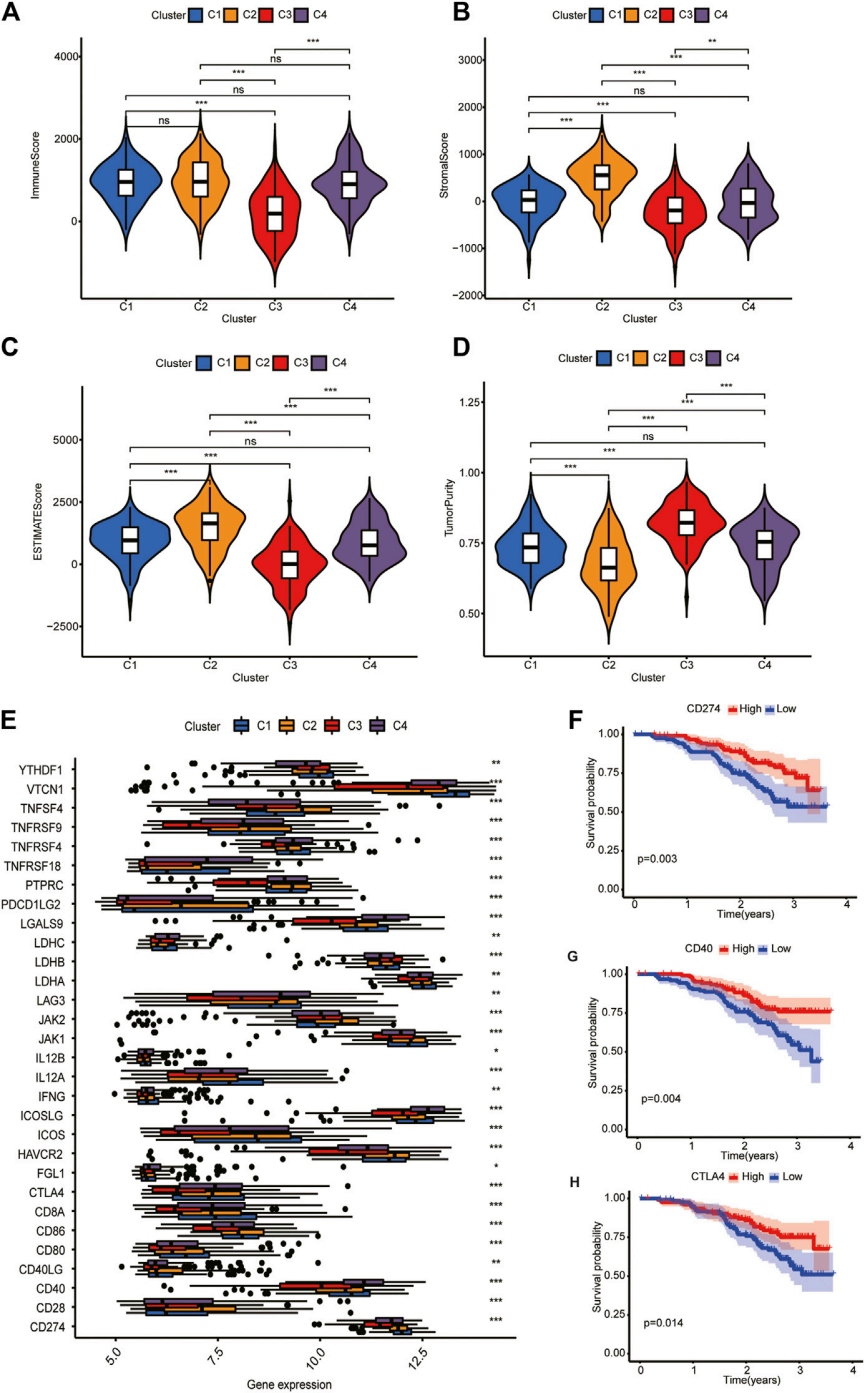
Tumor microenvironment scores and ICGs expression across three HCC subtypes. **(A,B)** Subtype II had the highest stroma scores and immune scores. **(C)** Tumor purity decreased in subtype III/IV/I/II. **(D)** Subtype II performed worst in OS analysis. **(E)** Differential expression analysis of 35 ICGs. Their expression was significantly different among different clusters. **(F–H)** Kaplan-Meier analysis of CD274, CD40, and CTLA4. Their upregulation is associated with a better prognosis.

According to tumor microenvironment score analysis, subtype II had the highest stromal scores and immune scores (Figures 4A, B), while tumor purity decreased in subtype III/IV/I/II (Figure 4C). Subtype II performed worst in OS analysis (Figure 4D). We will further explain this result after the risk model is established.

Differential expression analysis revealed that 30 ICGs were differentially expressed in the four subtypes (Figure 4E). Kaplan–Meier analysis of ICGs showed that upregulation of CD274, CD40, and CTLA4 was associated with better prognosis (Figures 4F–H). We will explain this result further in “*Discussion*” section.

### 3.7 Generation and quality evaluation of a prognostic risk scoring model

**FIGURE 5:**
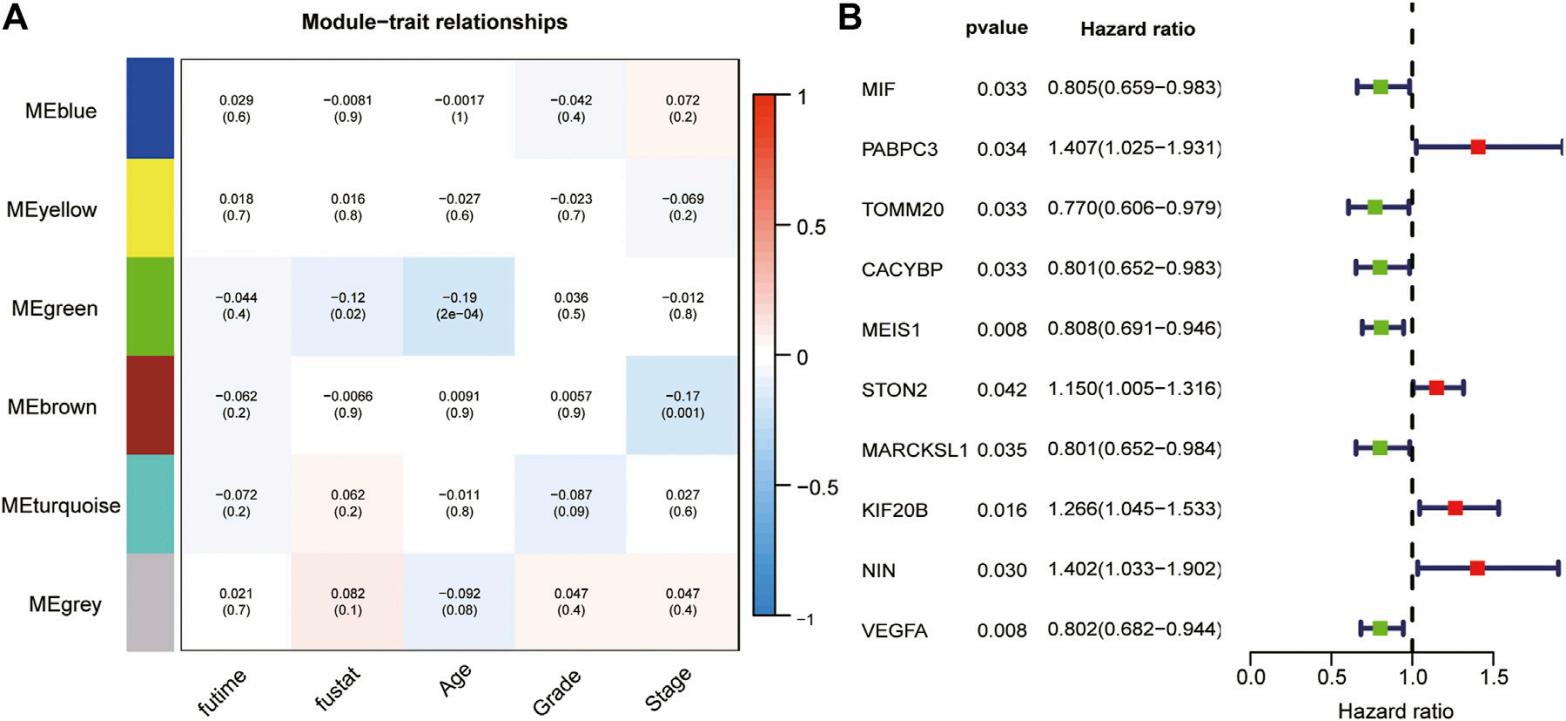
WGCNA and analyze significant genes by uniCox. **(A)** The brown module closely correlated OC stage (*p* = 0.001). They were therefore included in subsequent studies. **(B)** Ten prognosis-related DRGs were identified by univariate analysis (*p* < 0.05).

We took the intersection of the genes in TCGA and GSE140082 cohorts. A total of 1,089 DRGs were subjected to weighted correlation network analysis (WGCNA). With a soft threshold = 5 (Supplementary Figures S2A–C), DRGs were divided into six modules, and the brown module was highly correlated with the OV stage, which contained 250 genes (Figure 5A). Univariate analysis was performed for the DRGs in the module Brown based on prognostic correlation (*p* < 0.05). Eleven genes were screened out and incorporated into multivariate Cox regression analysis (Figure 5B). Finally, we established a prognostic risk scoring model that included nine genes and their relative risk coefficients. Using gene expression and risk factors, we obtained risk score = (−0.23018 × expression of MIF) + (0.38532 × expression of PABPC3) + (−0.25897 × expression of TOMM20) + (−0.18893 × expression of CACYBP) + (−0.35784 × expression of MEIS1) + (0.13692 × expression of STON2) + (0.42101 × expression of KIF20B) + (0.39011 × expression of NIN) + (−0.33416 × expression of VEGFA).

**FIGURE 6:**
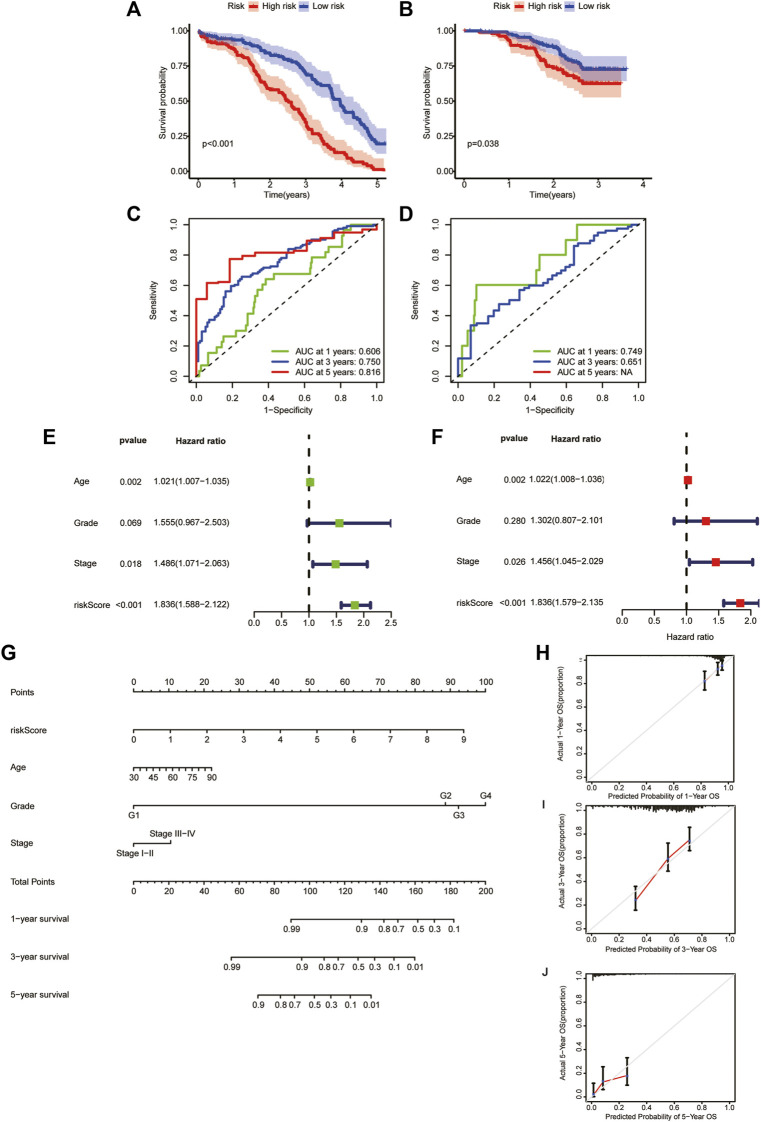
Generation and evaluation of a prognostic risk scoring model and a nomogram. **(A)** Survival analysis between high-risk group and low-risk group in the training sets. **(B)** Survival analysis between high-risk group and low-risk group in the validation sets. **(C)** Areas under ROC curve of the training sets to predict 1-, 3-, and 5-year OS. **(D)** Areas under ROC curve of the validation sets to predict 1-year and 3-year OS. **(E)** Univariate analysis of risk score and clinicopathological features. **(F)** Multivariate analysis of risk score and clinicopathological features. **(G)** A nomogram for predicting 1-year, 3-year, and 5-year OS. **(H–J)** The calibration curves for predicting 1-year, 3-year, and 5-year OS.

To verify the reliability of our model, we calculate the OS of the training and test sets through the model. The results showed that OS was significantly better in the low-risk group than in the high-risk group (Figures 6A, B). Additionally, the areas under the ROC curves for predicting 1-year, 3-year and 5-year OS were 0.606, 0.750 and 0.816 in the training set and 0.749 and 0.651 in the test set, respectively (Figures 6C, D) (due to the lack of the clinical data of test set samples, the 5-year OS could not be verified).

### 3.8 Establishment and quality evaluation of a nomogram

We performed univariate and multivariate analyses of TCGA cohorts (Figures 6E, F). The results showed that age, stage, and risk score significantly influenced patient outcomes. The older the patient is, the later the clinicopathological stage and the higher the RS, leading to a worse prognosis. Based on these factors, we constructed a nomogram to predict OS at 1, 3 and 5 years (Figure 6G). To assess the goodness of fit for the nomogram, we generated calibration curves predicting 1-, 3- and 5-year OS. The results showed that it fit well with the reference line (Figures 6H–J).

### 3.9 Hypoxia-driven immune escape in ovarian cancer

After the risk scoring model was established, we performed GSVA analysis on T cells in the high-risk and low-risk groups (Supplementary Figure S3). The results showed that the hypoxia pathway was significantly enriched in the high-risk group, while hypoxia-driven immune escape was significant in ovarian cancer (Johnson et al., 2021). Hypoxia promotes tumor production of interleukin (IL-10) to recruit regulatory T cells (Tregs) to the TME, thereby blocking the antitumor immune response. This result provided a possible explanation for the phenomenon that subtype II had the highest stromal scores and immune scores (lowest tumor purity) and a richest immune environment, but had the worst performance in OS analysis. (In section “Analysis of tumor microenvironment scores and ICGs expression across OV clusters”).

## 4 Discussion

### 4.1 Main findings

In this study, based on the analysis of scRNA-seq data, OV heterogeneity was further explored from the perspective of cell differentiation trajectory. We identified six ovarian cancer clusters and identified differentiation-related genes (DRGs). Then, ovarian cancer samples were divided into 4 DRG-based subtypes. There were significant differences in survival rates, clinical traits, tumor microenvironment scores, and the expression levels of ICGs among the subtypes. The DRGs were subjected to multivariate Cox regression analysis to generate a prognostic risk score (RS) model. Finally, we obtained a nomogram of the prognostic variable combination, including RS and clinicopathological characteristics, and predicted the 3-year and 5-year overall survival.

### 4.2 Strengths and limitations

The main strength of this study is to further explore the molecular typing for ovarian cancer from the perspective of cell differentiation trajectory. To the best of our knowledge, this idea has not been investigated in previous studies. In addition, we used multiple omics data for analysis. ScRNA-seq technology paves a new way to explore intratumoral heterogeneity within tumors. Based on the analysis of scRNA-seq data, genes related to cell differentiation trajectory were identified, and bulk RNA data were used for molecular typing. Finally, a risk scoring model is generated based on TCGA clinical data, and a visualization method is provided by using the nomogram.

However, limited by the scRNA-seq samples available on GEO, our study did not explore applicability in different histological types of ovarian cancer. Besides, due to the small size of the data sets from TCGA and GEO databases, the reliability and practicability of our risk scoring model should be further validated by large-scale clinical studies. What’s more, because the TCGA and GEO are repositories for pre-defined variables, some potentially relevant clinical variables are not accessible to us. The variables contained in our nomogram may not be comprehensive enough, even if it has exhibited a high degree of accuracy.

### 4.3 Interpretation

Ovarian cancer is highly heterogeneous, presenting with diverse morphology, natural history, and treatment responses. In recent years, the molecular characteristics of ovarian tumors have been studied extensively, and the biological and clinical subtypes of related diseases have been identified accordingly.

Classification is helpful in the diagnosis and treatment of tumors. The WHO divides epithelial ovarian cancer into five histological subtypes [including high-grade serous adenocarcinoma (70%), endometrioid adenocarcinoma (10%), clear cell adenocarcinoma (10%), mucinous adenocarcinoma (3%), and low-grade serous adenocarcinoma (<5%) (Pearce et al., 2012; Sung et al., 2014; Miller et al., 2017b)]. However, this single traditional classification is not a good predictor of prognosis. Singer, Shih et al. established a dualistic model to classify ovarian epithelial carcinoma as slow-growing type I tumor and fast-growing and highly invasive type II tumor (Singer et al., 2002; Singer et al., 2005). Type I tumors are characterized by mutations in many different genes, including KRAS, BRAF, PTEN and β-catenin (Rojas et al., 2016). Type II tumors grow rapidly and are highly aggressive but lack well-defined precursor lesions. Most of them are in the late stages at the beginning or soon after. However, this dualistic model is not perfect: the molecular genetic characteristics of each subtype within type I and type II are similar but not identical, so this classification method cannot be used as an independent factor to predict the prognosis of patients. In addition, high-grade serous carcinoma in type II is highly heterogeneous, and it is not clear whether there are other subsets of type II.

With the development of molecular biology and molecular diagnosis, more molecular typing methods for ovarian cancer have been proposed. In 2008, Tothill et al. determined the miRNA gene expression profile by chip and identified six subtypes by the k-means method (Tan et al., 2013). Tan et al. identified five molecular subtypes through functional genomics in 2013 (Kommoss et al., 2017). However, these methods have not been widely used in clinical practice. Here, ovarian cancer cells with different cell differentiation states were divided into 4 subtypes, and multiple omics data were used. Therefore, our study can effectively supplement the current molecular typing strategies for ovarian cancer.

It is worth mentioning that, for the strength and validity of our conclusions, we only selected serous ovarian cancer to study. Serous ovarian cancer is the most common type of ovarian cancer and the most malignant. After validation of clinical samples, we will gradually apply molecular typing methods to other histological types of ovarian cancer in subsequent studies. For the study of other small subtypes, the experimental analysis should be redesigned after excluding high-grade serous phenotype.

Further, in our study, DRGs are produced by malignant cells (epithelial cells). They may not reflect the specific differences and distinctions of malignant cells, because the presence of other cell types may affect the risk scoring (e.g., CAFs, T cells, etc.). However, it does not affect the positive role of DRGs in prognosis prediction in clinical practice. Just like the subset C2’s example, its population seems to have reduced survival but it also has higher stromal content. It showed that even if the malignant cells are low-abundance, it still has an effect on poor prognosis (the status of malignant cells matter more). This is similar to the TCGA “Mesenchymal” subtype. However, for patients with ovarian cancer, we think it is better to describe the status of malignant cells and not as indirectly as mesenchyme.

In the relevant studies of immune checkpoint genes, it is generally believed that the upregulation of immune checkpoint genes will lead to the suppression of immune function, the increase of tumor infiltration and poor prognosis. In our study, survival analysis of CD274, CD40, and CTLA4 showed that their upregulated expression all led to better prognosis. To verify this result, we used GEPIA2 (Tang et al., 2019) to analyze the OV samples in TCGA database (Supplementary Figure S1). The results showed that their upregulation also led to a better prognosis. Similar conclusions in other cancers were reached by Hu et al. (2021). We think this is due to the different expression patterns of ICGs, which may affect immune checkpoint blockade (ICB) response patterns and lead to different prognosis. We also hope that these genes will help to understand the mechanisms of ICGs in ICB signal pathways and other anticancer treatments in future studies.

Our study found that the survival curves of different molecular types were significantly different, indicating that this method can be used to predict patient OS. A risk scoring model was established by multivariate Cox regression analysis. Most of the genes in the model have been identified to play important roles in the progression and prognosis of ovarian carcinoma. For example, macrophage migration inhibitory factor (MIF) can inhibit the activation of EGFR, while EGFR can promote the growth of various tumors and has been identified as a key therapeutic target of epithelial ovarian cancer (Sheng and Liu, 2011; Zheng et al., 2015). PABPC3 is a mutated driver gene of many cancers (Erinjeri et al., 2018; Chen et al., 2021). STON2 modulates stem-like properties in ovarian cancer cells, which are highly associated with poor prognosis and invasion (Sun et al., 2017; Xu et al., 2018). MEIS1 triggers chemokine expression and involvement in CD8^+^ T-lymphocyte infiltration in early-stage ovarian cancer (Karapetsas et al., 2018). These genes are considered important molecular markers.

## 5 Conclusion

In our study, single-cell data were classified and presented. OV samples were divided into four molecular types based on cell differentiation trajectories, which differed significantly in expression profile, clinical features and prognosis. Based on nine DRGs, a prognostic risk score model was generated, which can be a good predictor of patient OS. Further, we used the model to classify the samples, and hypoxia-driven immune escape pathways were found to be enriched in the high-risk group. In conclusion, this study highlights the importance of cell differentiation for molecular typing of OV, and provides new ideas for predicting prognosis and potential immunotherapy of OV patients.

## Data Availability

The original contributions presented in the study are included in the article/Supplementary Material, further inquiries can be directed to the corresponding authors.
