# Supplementary figures and images for: Molecular typing and prognostic risk models for ovarian cancer: a study based on cell differentiation trajectory
Source: Front Cell Dev Biol. 2023 Aug 31;11:1131494. doi: 10.3389/fcell.2023.1131494 (PMC10500593; doi:10.3389/fcell.2023.1131494)

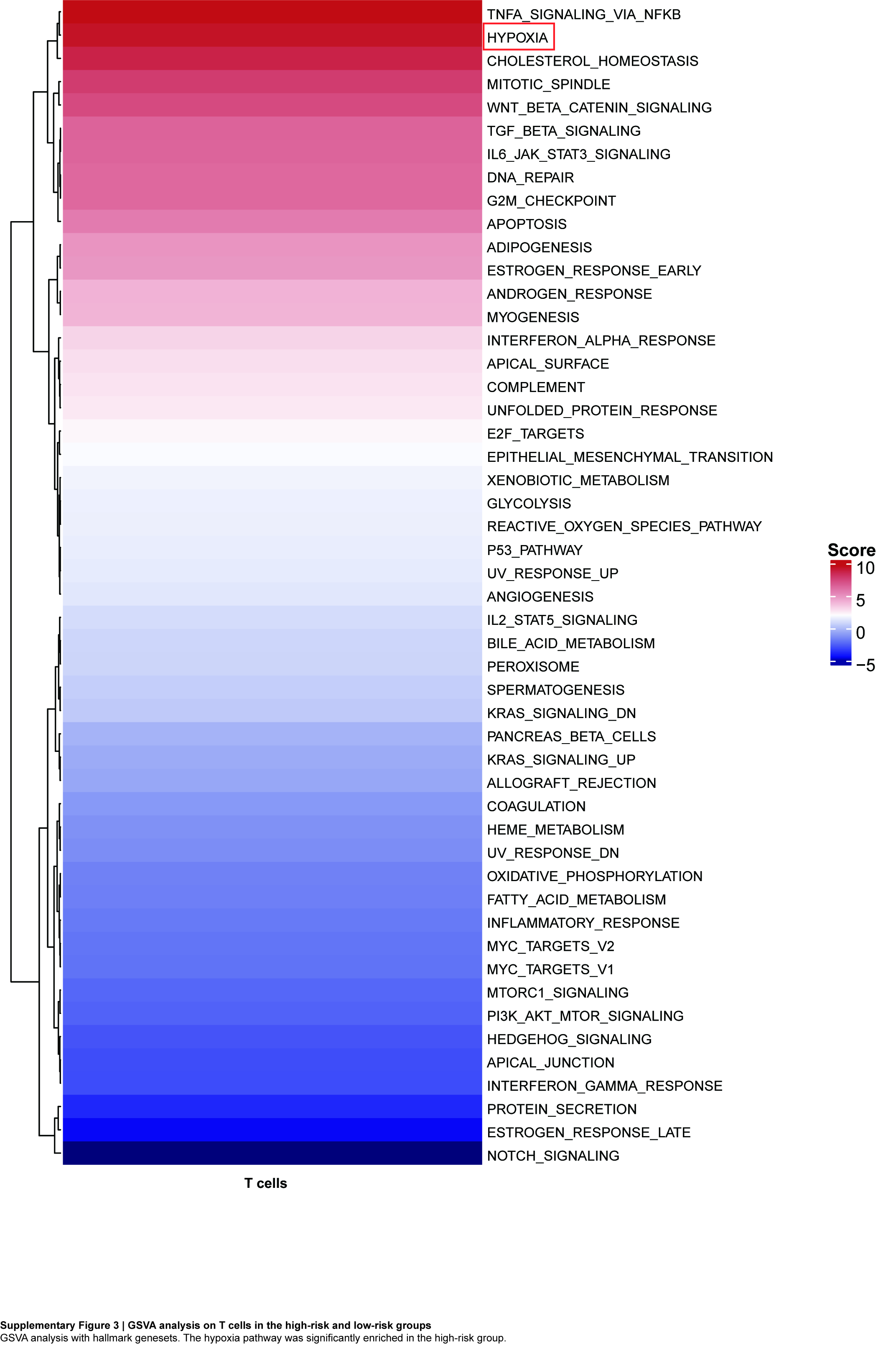

Supplement: Supplementary file 1 [file Image3.TIF]

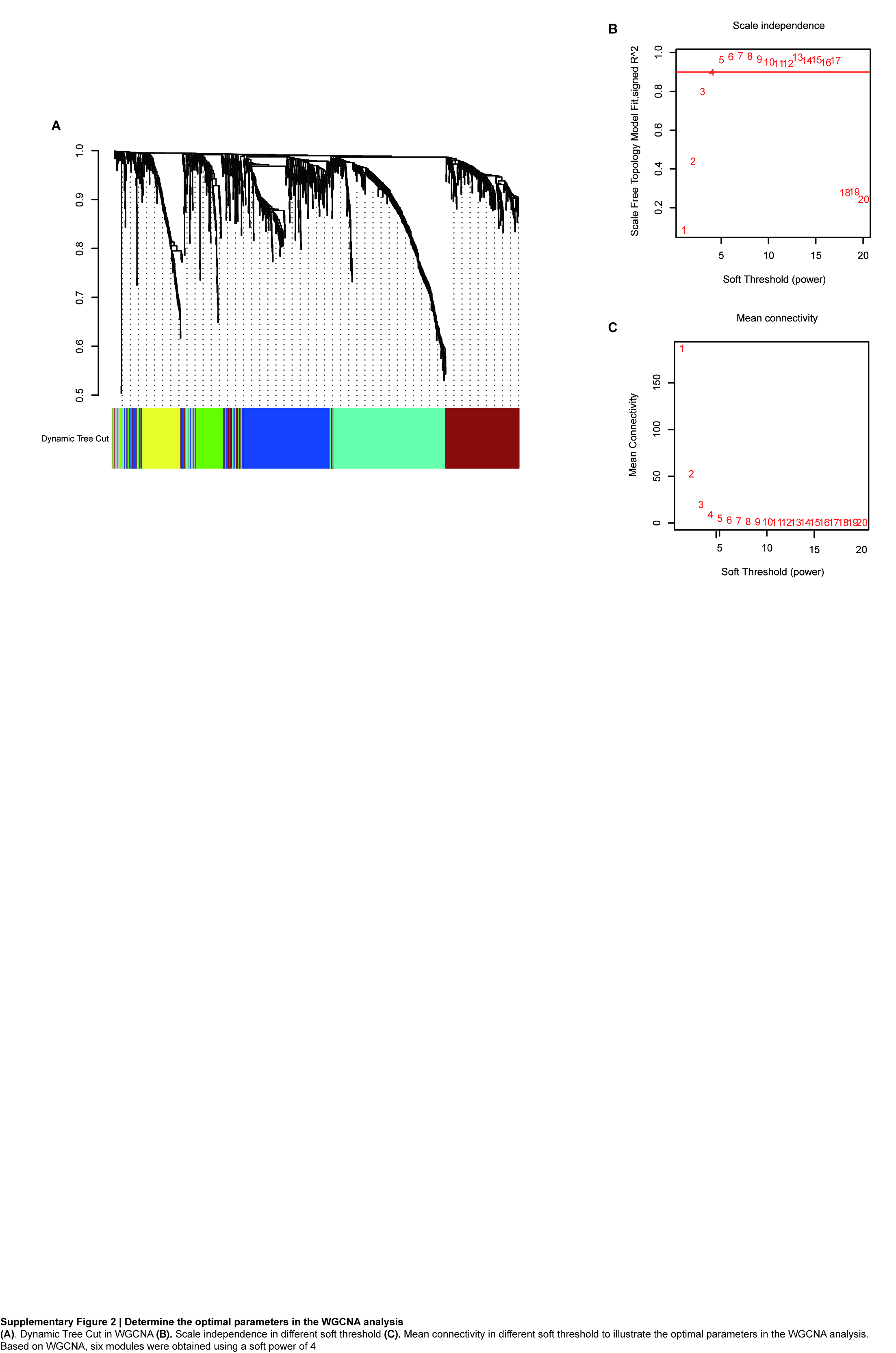

Supplement: Supplementary file 3 [file Image2.TIF]

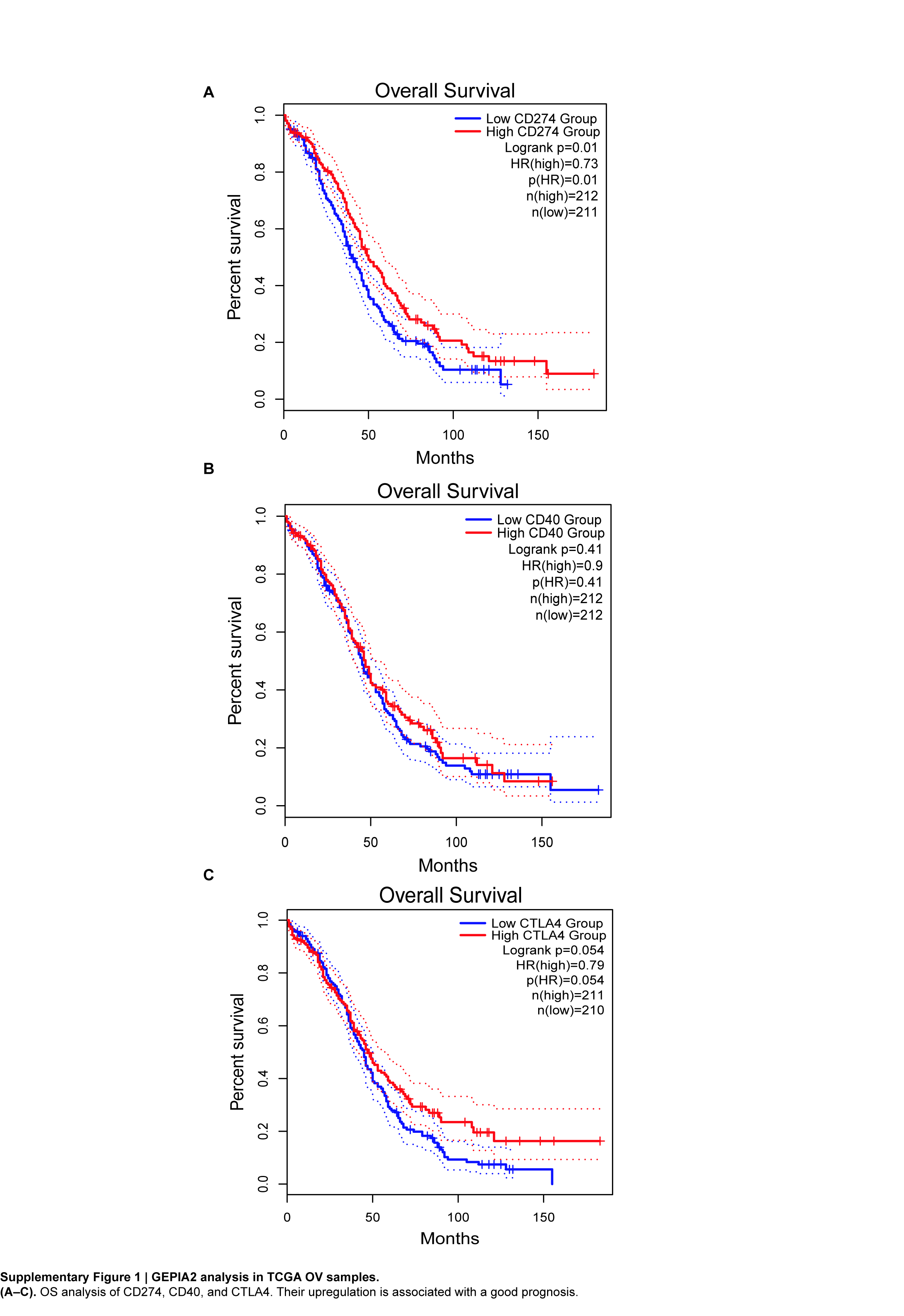

Supplement: Supplementary file 4 [file Image1.tif]
